# Isolation of Circulating Tumor Cells from Seminal Fluid of Patients with Prostate Cancer Using Inertial Microfluidics

**DOI:** 10.3390/cancers14143364

**Published:** 2022-07-11

**Authors:** Alexey S. Rzhevskiy, Alina Y. Kapitannikova, Steven A. Vasilescu, Tamilla A. Karashaeva, Sajad Razavi Bazaz, Mark S. Taratkin, Dmitry V. Enikeev, Vladimir Y. Lekarev, Evgeniy V. Shpot, Denis V. Butnaru, Sergey M. Deyev, Jean Paul Thiery, Andrei V. Zvyagin, Majid Ebrahimi Warkiani

**Affiliations:** 1 Institute of Molecular Theranostics, Sechenov First Moscow State Medical University, 119991 Moscow, Russia; kapitannikova_a_yu@staff.sechenov.ru (A.Y.K.); karashaeva_t_a@staff.sechenov.ru (T.A.K.); biomem@mail.ru (S.M.D.); bchtjp@nus.edu.sg (J.P.T.); majid.warkiani@uts.edu.au (M.E.W.); 2 Institute for Urology and Reproductive Health, Sechenov University, 119146 Moscow, Russia; taratkin_m_s@staff.sechenov.ru (M.S.T.); enikeev_d_v@staff.sechenov.ru (D.V.E.); lekarev_v_yu@staff.sechenov.ru (V.Y.L.); shpot_e_v@staff.sechenov.ru (E.V.S.); butnaru_d_v@staff.sechenov.ru (D.V.B.); 3 School of Biomedical Engineering, University of Technology Sydney, Sydney 2007, Australia; steven.a.vasilescu@student.uts.edu.au (S.A.V.); sajad.razavibazaz@student.uts.edu.au (S.R.B.); 4 Department of Urology, Medical University of Vienna, 1090 Vienna, Austria; 5 Molecular Immunology Laboratory, Shemyakin & Ovchinnikov Institute of Bioorganic Chemistry, Russian Academy of Sciences, 117997 Moscow, Russia; 6 Research Centrum for Oncotheranostics, Research School of Chemistry and Applied Biomedical Sciences, Tomsk Polytechnic University, 634050 Tomsk, Russia; 7 Bio-Nanophotonic Laboratory, Institute of Engineering Physics for Biomedicine (PhysBio), National Research Nuclear University “MEPhI”, 115409 Moscow, Russia; 8 Guangzhou Laboratory, Bioisland, Guangzhou 510320, China; 9 MQ Photonics Centre, Macquarie University, Sydney 2109, Australia

**Keywords:** microfluidics, liquid biopsy, circulating tumor cells, prostate cancer, seminal fluid

## Abstract

**Simple Summary:**

Prostate cancer (PCa) is notoriously difficult to diagnose owing to the lack of reliable biomarkers and the invasiveness of obtaining a tissue biopsy from the prostate. As an alternative, we developed a liquid biopsy technique, based on isolating tumor cells from semen samples via a microfluidic device. To optimize the device, we first attempted to recover PCa cells from semen samples spiked with PCa cell lines, achieving an average efficiency of >87% cell recovery at the chosen flow rate. We then transitioned to a clinical setting using semen samples from PCa patients. The yield of isolated clinical PCa cells varied between 67 and 307 cells per mL of semen (in 15 cancer patients). These cells were stained and compared to the standard prognostic parameters such as Gleason score and PSA serum level. This study presents a potential liquid biopsy technique to augment the existing diagnosis and prognosis of PCa.

**Abstract:**

Prostate cancer (PCa) diagnosis is primarily based on prostate-specific antigen (PSA) testing and prostate tissue biopsies. However, PSA testing has relatively low specificity, while tissue biopsies are highly invasive and have relatively low sensitivity at early stages of PCa. As an alternative, we developed a technique of liquid biopsy, based on isolation of circulating tumor cells (CTCs) from seminal fluid (SF). The recovery of PCa cells from SF was demonstrated using PCa cell lines, achieving an efficiency and throughput as high as 89% (±3.8%) and 1.7 mL min^−1^, respectively, while 99% (±0.7%) of sperm cells were disposed of. The introduced approach was further tested in a clinical setting by collecting and processing SF samples of PCa patients. The yield of isolated CTCs measured as high as 613 cells per SF sample in comparison with that of 6 cells from SF of healthy donors, holding significant promise for PCa diagnosis. The correlation analysis of the isolated CTC numbers with the standard prognostic parameters such as Gleason score and PSA serum level showed correlation coefficient values at 0.40 and 0.73, respectively. Taken together, our results show promise in the developed liquid biopsy technique to augment the existing diagnosis and prognosis of PCa.

## 1. Introduction

Prostate cancer (PCa) is one of the most common cancers worldwide and remains the third leading cause of cancer death in men [1]. Although PCa is common, there is still controversy regarding the utility of the existing screening and diagnostic methods. Prostate-specific antigen (PSA) blood tests in combination with invasive tissue biopsy are regarded the gold standard for early detection of PCa [2] despite the following shortcomings. The specificity of the PSA blood test is known to be relatively low, especially in the case of the localized form of the disease. At the same time, while the sensitivity of the prostate tissue biopsy is moderate, it has to be accompanied by the needle-guiding radiological imaging and carries the risk of potential side-effects including local abscesses and bleeding [3]. For the past decade, detection and assaying of circulating tumor cells (CTCs) has advanced to allow diagnosis and prognosis of diverse cancers, including PCa [4]. However, in the case of localized PCa, CTCs are relatively rare, measuring as few as several cells per milliliter of the blood [5,6,7,8,9,10,11]. Moreover, PCa CTC isolation remains cumbersome using the existing liquid biopsy techniques. As a result, the isolation and assaying of PCa CTCs from the peripheral blood is considered inadequate for the early detection of PCa [12].

It has been reported that CTCs released from the primary tumor can escape to the blood stream and urine, while the escape pathway into the prostatic ducts has been largely overlooked. The detection of CTCs in urine reported to date [13,14,15] speaks in favor of this possibility. An increase in the PCa biomarkers, including PCa antigen 3, in urine has been observed [16] during the digital rectal examination, which was known to squeeze some prostatic fluid into the urethra. One can conjecture that biomarkers detected in the urine originate from the prostatic gland [17]. Therefore, the seminal fluid (SF) naturally abundant with prostatic fluid may represent an alternative to the blood and urine.

In 1996, Gardiner et al. described the presence of PCa CTCs in the ejaculate of PCa patients [18]. In this study, the SF was centrifuged and the resultant pellet was cast into smears, followed up by microscopic examination to detect CTCs. Atypical prostatic cells in the SF specimens were confirmed in 75% of the cohort. The CTC detection in the ejaculate smears was hindered by the overwhelming background of sperm cells measured in hundreds of millions per milliliter of the SF. Flow cytometry of the processed SF has allowed augmenting the procedure of the identification and counting of PCa CTCs but at the expense of compromising cell viability and wasting rare cells [19]. Therefore, an optimized automated method of isolating CTCs from SF is required to discriminate CTCs from the unwanted background of sperm cells and seminal plasma debris and maintain the isolated cell viability. To this aim, we adopted the inertial size-based microfluidic technology.

The inertial microfluidics has demonstrated excellent performance for CTC isolation and assaying from the blood of cancer patients [20,21,22,23,24,25,26] as well as CTC isolation from the urine of PCa patients [15]. However, this is the first time, to the best of our knowledge, that CTC isolation from the SF of patients with an early localized form of PCa by means of inertial microfluidics is reported and demonstrates significant diagnostic and prognostic potential. This technique is believed to complement the existing gold-standard PCa diagnostics suite. Purification of sperm cells for cryopreservation is another worthwhile possibility afforded by our technology.

## 2. Materials and Methods

### 2.1. Microfluidic Device and Its Fabrication

The deployed microfluidic device architecture has been reported by Warkiani et al. [20,24,25,26], and was adopted for the isolation of CTCs from the SF. A microfluidic chip was structured as two polydimethylsiloxane (PDMS) layers (Sylgard 184 from Dow Corning, Midland, MI, USA) made by mixing liquid PDMS and curing agent in a 10:1 ratio (*w*/*w*). The top layer of the device fabricated by employing the previously reported micromolding process was bonded to a flat layer of PDMS following oxygen plasma treatment for 2 min (Harrick Plasma, Ithaca, NY, USA); 1.5 mm holes were punched at the beginning and ends of the microfluidic channel and 1.5 mm tygon tubes were inserted into each inlet/outlet.

### 2.2. Cell Culture

The proof-of-concept experiments were performed using DU145, PC3, and LNCaP PCa cell lines. These cell lines were cultured under standard conditions in a humidified incubator (Panasonic, Seoul, Korea) at 37 °C, in 5% CO_2_. DU-145 and PC-3 cell lines were cultured in RPMI-1640 medium (Thermofisher Scientific, Waltham, MA, USA) with Glutamax supplement (Gibco, Jenks, OK, USA), 10% heat-inactivated, 0.22 µm filtered fetal bovine serum (Life Technologies, Inc., Carlsbad, CA, USA), and 1% penicillin–streptomycin (Life Technologies, Inc.). For LNCaP cell line, an RPMI-1640 medium (Thermofisher Scientific) supplemented with 10% heat-inactivated, 0.22 µm filtered fetal bovine serum (Life Technologies, Inc.), 1% insulin-transferrin (Paneco, Moscow, Russia), 1% HEPES pH 7.2–7.5 (Paneco), 1% nonessential amino acid solution (Gibco, USA), and 1% gentamycin (Paneco) was used. RAW264.7 cell line, used as a negative control, was cultivated by using DMEM-F12 (Paneco) media with 1% penicillin (Invitrogen, Waltham, MA, USA), 100% streptomycin (Invitrogen, USA), and 10% bovine serum albumin (BSA) (Invitrogen, USA). T-25 and T-75 filtered flasks (Corning, NY, USA) were used for subculturing of the cell lines. Cell dissociation was performed using 0.25% (*w*/*v*) Trypsin-0.53 mM EDTA solution (Thermofisher Scientific, USA). Replating was carried out at a 1 to 6 ratio. Cell culture medium was renewed every 2 or 3 days. Cell lines were STR-profiled for authenticity.

### 2.3. Proof-Of-Concept Experiments

The ability of the microfluidic chip to isolate PCa CTCs from the SF was initially evaluated using SF samples, obtained from healthy volunteers of young males under 30 years of age, separately spiked with a predetermined number of DU-145, LNCaP, or PC3 cells at approximately 1000 cells per standard volume (2–3 mL) of healthy male SF. The chosen prostate cancer cell lines are represented by the cells at higher than 13 µm in mean diameter, which is one of the basic requirements for the tumor cells to be successfully isolated with the developed technique. Considering the fact that the CTCs, which could have been previously isolated from blood, have a diameter as high as 16.9 µm [27], the chosen cell lines are adequate for being used as the model of CTCs.

For proper characterization, the tumor cells were first stained with 4′,6-diamidino-2-phenylindole (DAPI) prior to SF spiking. Separation was then performed at flow rates of 1.1, 1.3, 1.5, 1.7, 1.9, and 2.1 mL/min to briefly characterize an ideal flow rate for further testing. As a control, the same number of PCa cells were spiked into the same volume of Dulbecco’s phosphate-buffered saline (DPBS) and processed across the same flow rates for comparison. To decrease viscosity of SF samples, the samples were kept for 30 min in a humidified incubator (Panasonic) at 37 °C prior to the addition of the tumor cells. The sample was then transferred to a 15 mL falcon tube, centrifuged at 1000 rpm for 7 min and the supernatant was replaced with 5 mL of DPBS. Then, the solution was processed using a microfluidic chip with a syringe pump mounted at the device input pumping tested fluid (Baoding Shenchen Precision Pump Co., Ltd., Baoding, China) connected with Tygon tubing (0.02-inch inner diameter, 0.06-inch outer diameter). The flow rate was optimized as 1.7 mL/min. The microfluidic chip outlet was split into ‘target’ and ‘waste’ fractions, where the target fraction contained most of the tumor cells (DU-145, LNCaP, or PC3), while the waste fraction contained most of the sperm cells. The mixture of cells in the target fraction was fixed with 4% paraformaldehyde (PFA) (ThermoFisher Scientific) solution in DPBS for 10 min, and therefore prepared for antibody labelling. After fixation, the solution was centrifuged at 1000 rpm and the supernatant was replaced with 5 mL of DPBS. Furthermore, a potential of additional processing of the target fraction through the microfluidic chip was assessed in terms of the separation efficiency of the residual sperm cells. All the experiments were repeated 5 times.

### 2.4. Clinical Samples

The second component of this study included the collection and processing of SF samples obtained from PCa patients and healthy volunteers, which was performed under the ethical approval provided by Sechenov University Local Ethics Committee (extraction from protocol Nos. 17–19). The samples were collected at Sechenov University Clinical Hospital No. 2, Moscow, Russia. All donors provided written informed consent for collection of their SF samples and provision of their clinical data. The study was conducted in accordance with the Declaration of Helsinki (2013). In total, 15 patients with diagnosed localized PCa and 15 healthy volunteers, represented by young men under 30 years without any prostatic diseases, provided SF samples. The SF samples were collected and analyzed in a non-blinded manner. SF samples were collected in sterile collection cups, and ranged between 0.5 mL and 3.5 mL in volume. Samples were transported from the hospital to the testing lab in a thermal container for microfluidic processing and further analysis. Each SF sample was incubated for 30 min in a humidified incubator (Panasonic) at 37 °C for liquefaction of the SF. Then, each sample centrifuged at 1000 rpm for 7 min and the pellets were resuspended in 5 mL of DPBS. After, the cells were fixed with 4% PFA solution in DPBS for 10 min, centrifuged again, and resuspended in 5 mL of DPBS. The solution was then processed using the microfluidic chip connected to the syringe pump (Baoding Shenchen Precision Pump Co., Ltd.) at a flow rate of 1.7 mL/min.

### 2.5. Immunofluorescence Staining

An antibody panel composed of the primary antibodies to such antigens as cytokeratins (CK), prostate-specific membrane antigen (PSMA), and Glypican 1 (GPC-1) were applied for identifying CTCs isolated from the patients’ samples. The CK is a standard antigen used in detecting CTCs in liquid biopsy, and liquid biopsy of PCa in particular [28]. The PSMA has also been proven effective for PCa tumor cell detection [29]. At the same time, GPC-1 antigen has recently been proven specific for PCa tumor cells [30]. Thus, combination of the chosen markers justifies high specificity of the antibody panel in identifying PCa CTCs.

For the proof-of-concept experiments using cultured PCa cell lines, collected fractions were centrifuged onto four adhesive glass slides (ThermoFisher Scientific, Brisbane, Australia) using a Thermo Scientific™ Cytospin™ 4 Cytocentrifuge (ThermoFisher Scientific).

Immunocytochemistry labeling panel consisted of the following antibodies: primary rabbit mAb IgG to PSMA (Abcam, ab76104) with corresponding Alexa Fluor 568 secondary Goat anti-rabbit IgG H&L (Abcam, ab175695), primary mouse mAb IgG to pan-cytokeratin (Abcam, ab 86734) with corresponding Alexa Fluor 488 secondary Goat anti-mouse IgG H&L (Abcam, ab150117), and recombinant mAb to Glypican 1/GPC1 directly conjugated with Alexa Fluor 647 (ab237290). Antibodies were tested and titrated individually and in combination on each PCa cell line. After isolation, the cells were fixed with 4% PFA permeabilized with 0.2% Tween-20 in PBS and incubated with blocking buffer with 10% goat normal serum and 10% rabbit normal serum (before incubation with anti-GPC1 mAb). The ICC staining was performed in sequence. After incubations with the primary and secondary antibodies, the cells were mounted with a ProLong Gold Anti-Fade Mountant (Abcam, Cambridge, UK) containing DAPI, covered with a coverslip and counted under a fluorescence microscope (Zeiss Axio Imager Z2 Upright Microscope, Oberkochen, Deutschland). The same number of DU-145 (or PC3, or LNCaP) cells as the one added to the SF sample were deposited onto the glass slide and labelled with anti-CK+AlexaFluor488 antibodies, then mounted with a ProLong Gold Anti-Fade Mountant (Abcam, Cambridge, UK) containing DAPI, covered with a coverslip, and counted under a fluorescence microscope (Zeiss Axio Imager Z2 Upright Microscope).

Sequential labelling was employed to identify isolated PCa CTCs in the clinical samples processed by using the microfluidic chip. After isolation, CTC samples were fixed with 4% Paraformaldehyde (Sigma-Aldrich, Saint Louis, MO, USA), 1.00496) for 15 min at room temperature. To stop protein cross-linking, slides were 3× times washed in 1× ice-cold PBS. Permeabilization was performed with 0.2% Tween-20 (Abcam, ab128987) in 1× PBS at room temperature. After that, slides were washed three times in 1x PBS. To reduce unspecific binding, a blocking step was performed with 10% normal goat serum (Abcam, ab7481) in 1× PBS for 1 h at room temperature.

Then, samples were incubated with recombinant primary rabbit mAb to PSMA (Abcam, ab76104, dilution 1:50) overnight at +4 °C in humidity chamber. After that, slides were washed 3× times × 5 min in 1× PBS with 0.05% Tween 20 (prepared from Abcam, ab128987). Samples were incubated with Alexa Fluor 568© secondary Goat anti-rabbit IgG H&L (Abcam, ab175695, dilution 1:1000) for 1 h in humidity chamber at room temperature. After that, slides were washed 3× times × 5 min in 1× PBS with 0.05% Tween 20 (prepared from Abcam, ab128987).

Blocking step was repeated with 10% normal goat serum in PBS for 1 h at room temperature (Abcam, ab7481). Samples were incubated with primary mouse mAb to pan-cytokeratin (Abcam, ab 86734, dilution 1:100) overnight in humidity chamber at +4 °C, washed 3× times × 5 min in 1× PBS with 0.05% Tween 20 (Abcam, ab128987) and incubated with corresponding Alexa Fluor 488© secondary Goat anti-mouse IgG H&L (Abcam, ab150113, dilution 1:1000) for 1 h in humidity chamber at room temperature. Samples were washed 3× times × 5 min in 1× PBS with 0.05% Tween 20 (Abcam, ab128987).

At the next step, blocking with 10% normal rabbit serum (Abcam, ab166640) in 1x PBS for 1 h at room temperature was performed. Following incubation with the fluorophore-conjugated secondary antibody, normal rabbit serum was used to block open binding arms of the secondary goat anti-rabbit IgG, preventing capture of the next labeled primary antibody. Finally, samples were incubated with Alexa Fluor 647© rabbit mAb to Glypican 1/GPC1 (Abcam, ab237290, dilution 1:100) overnight at +4 °C in humidity chamber. After washing 3× times × 5 min in 1× PBS with 0.05% Tween 20 (Abcam, ab128987), slides were rinsed with bi-distilled water to prevent formation of PBS crystals and mounted with coverslips using ProLong™ Gold Antifade Mountant with DAPI (Thermofisher Scientific, Invitrogen, P36931. After the staining procedure was finished, samples were observed under laser scanning microscopy immediately. Otherwise, slides were covered in aluminum foil and for a short time stored at +4 °C in darkness. All the solutions were freshly prepared and filtered before the experiments. Antibodies were diluted in 1x PBS with 1% BSA (Sigma-Aldrich, A2153) right before the incubation.

### 2.6. Cell Enumeration and Data Analysis

Laser-scanning confocal microscope Zeiss LSM880 (ZeissAG, Oberkochen, Germany) supported by Zen Black software (version 3.1), was employed to image glass slides with tested specimens. To establish a benchmark for identification and enumeration of the isolated CTCs, healthy SF samples were spiked with approximately 1000 PC3 or DU-145 cells, processed by the chip, and immunocytostained as described above. Images of the immunocytostained cells were then obtained using laser scanning microscope Zeiss LSM880 and Zen Black software. The acquired fluorescence signal intensity of cells was quantified by using Fiji ImageJ software. Furthermore, the mean (±SD) value of the signal intensity was measured and assigned as a threshold for fluorescence signal intensities from different fluorescent wavelengths. The isolated cells were assigned as PCa cells if the signal intensity was above the established thresholds for all three fluorescence channels (PSMA+, CK+, GPC-1+). In addition, to evaluate the potential diagnostic and prognostic value of the technique, a correlation between the numbers of the isolated CTCs and PSA serum levels or Gleason score (GS) was estimated.

## 3. Results

### 3.1. Recovery of CTCs from Spiked SF

**Figure 1:**
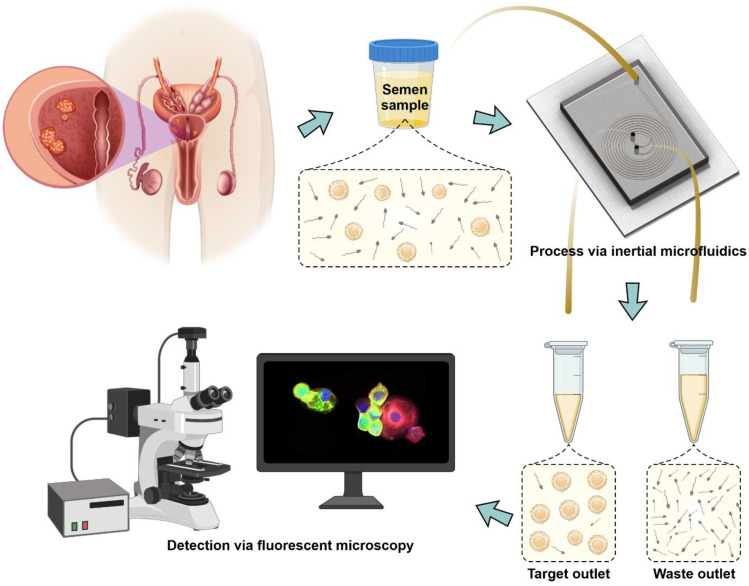
Schematic illustration of the technique for microfluidic isolation of CTCs from SF, with further detection of the isolated CTCs.

The primary aim of this study was to characterize the performance of a microfluidic cell separation device in terms of its efficiency in isolating PCa CTCs (Figure 1).

**Figure 2:**
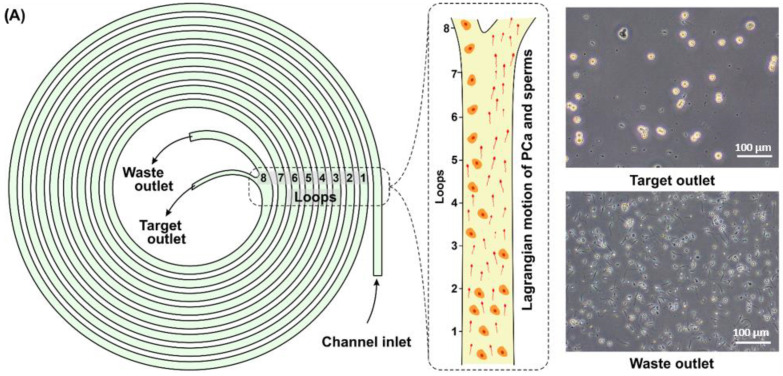

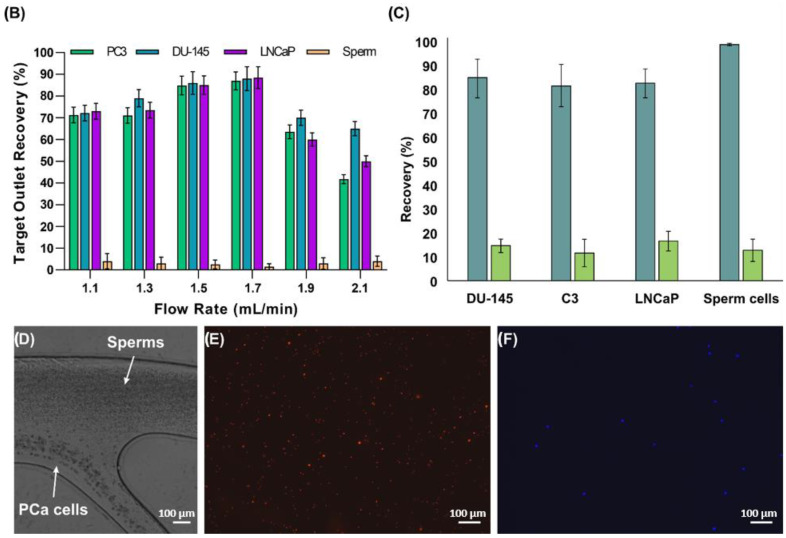
(**A**) Schematic representation of the spiral microfluidic channel for isolating CTCs from the SF. First, a preliminary prepared SF sample from a PCa patient is provided into the microchannel through the inlet under a continuous fluid flow at 1.7 mL/min. As a result, the SF sample is divided into target fraction containing most of the tumor cells and waste fraction containing most of the sperm cells. (**B**) Recovery rates of spiked PC3, DU-145, LNCaP cells, and sperm cells at different flow rates. (**C**) Recovery rates of spiked PCa tumor cells from three different cell lines (DU-145, PC3, and LNCaP) and sperm cells, after first and second runs of the device. The first run of the device implied the initial processing of the SF sample through the microfluidic chip. Thus, after the first run, it was possible to isolate more than 82% on average of the tumor cells, while more than 99% of the sperm cells could have been removed. The second run of the device implied processing of the target fraction. The processing of the target fraction resulted in a loss of the tumor cells and removal of the sperm cells in comparable percentages, at an average of 15% from the total number of tumor and sperm cells presenting in the target fraction. (**D**) High-speed camera image of CTC isolation from SF at the bifurcation point of the microfluidic channel. (**E**,**F**) Fluorescent images of target and waste fractions, respectively, showing a high concentration of DU-145 cells (blue dots) in the target fraction, and primarily sperm cells (red dots) in the waste fraction.

The inertial microfluidic device comprised of a microfluidic channel (Figure 2A) with a trapezoidal cross-section: one inlet and two outlets separated by a bifurcation of the channel (Figure 2D). To determine the optimal flow rate for CTC isolation from SF cell suspensions, a range of flow rates were tested using healthy SF samples spiked with PC3, DU-145, and LNCaP cells in separate experiments (Figure 2B). The resultant cell suspensions from the target (mostly tumor cells) and waste (mostly sperm cells) outlets can be seen in Figure 2A (a video of the high-speed cell separation is available in Video S1). The flow rates ranging from 1.1 to 2.1 mL/min were tested. As a control, the same PCa cell lines were also spiked into DPBS and exhibited comparable separation efficiency to cell isolation from SF (2B and Figure S3). The device performance, operated at flow rates of more than 1.7 mL/min, exhibited a distinct drop in the isolation efficiency of tumor cells. At the same time, the device performance operated at flow rates of less than 1.5 mL/min was similar to that of 1.7 mL/min but the operation rate was slower. The flow rate of 1.7 mL/min was chosen as the optimal flow rate since it produced the highest average recovery of tumor cells (87 ± 4.5% for PC3, 88 ± 4.6% for DU-145, and 89 ± 3.8% for LNCaP). The sperm recovery rates were above 97% for all the tested flow rates. As a result, the majority of cancer cells were isolated and presented into the target fraction (Figure 2E), while the majority of the sperm cells were separated and presented into the waste fraction (Figure 2F). The content of spiked tumor cells collected from the waste outlet is presented in the Figure S2.

Furthermore, DU-145, PC3, and LNCaP cells lines were individually spiked into healthy SF samples and processed at a flow rate of 1.7 mL/min. The recovery rates were 85 (±8)%, 82 (±9)% and 83 (±6%) for DU-145, PC3, and LNCaP cells, respectively (Figure 2C). The recovery performance was comparable across the tested cell lines and demonstrated a similar level of recovery at more than 82% on average after a single processing with the microfluidic device, while more than 99% (±0.7%) of the sperm cells could have been removed. The additional processing of the target fraction resulted in a loss of 15 (±3)%, 12 (±6)%, and 17 (±4)% of DU-145, PC3, and LNCaP cells, respectively, and additional removal of 13 (±5)% of the sperm cells, calculated from the total number of the cells presenting in the target fraction. Thus, the additional processing of the targeted fraction resulted in a loss of the cancer cells and removal of the sperm cells in comparable percentage number of 15%. Therefore, additional purification of the target fraction from sperm cells by means of the second processing was deemed unreasonable. As a result, each SF sample was processed through the microfluidic chip just once.

### 3.2. Isolation of Putative CTCs from the Patients’ SF

SF samples from the group of 15 patients with diagnosed PCa at its localized stage, and from 15 healthy males aged from 18 to 30 years as a control group, were acquired. Cells collected from the target outlet of the device were considered CTCs if they were more than 13 µm in mean diameter; featured the cytoplasm area at more than 232 µm^2^ determined from measurements of DU-145 and PC3 cells of the mean area of (284 ± 52) µm^2^; had the cell nucleus mean area at more than 92 µm^2^, measured from DU-145 and PC3 cells; and were positive for three antigens: CK (CK+), PSMA (PSMA+), and GPC-1 (GPC-1+). CK+, PSMA+, and GPC-1+ are defined as the cells exhibiting a fluorescence signal of more than 1572, 1589, and 1150 arbitrary units, respectively. The confirmation values were based on the mean fluorescent intensities of DU-145 and PC-3 cells measured as 1771 ± 199, 1867 ± 278, and 1423 ± 273 for the corresponding antigens, respectively.

**Figure 3:**
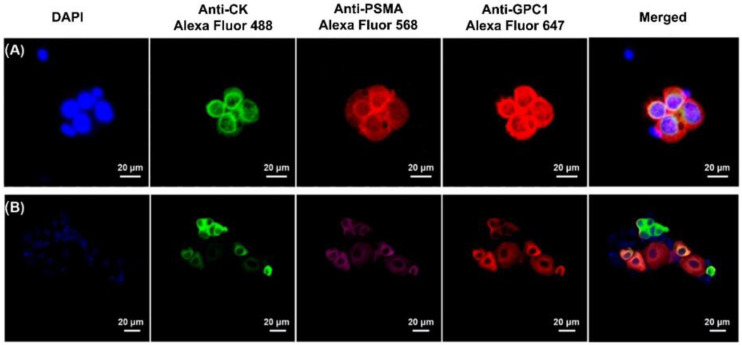

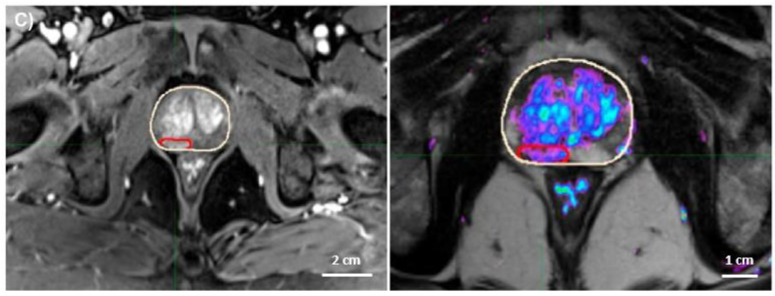
(**A**,**B**) The putative tumor cells overexpressing all three antigens of interest (PSMA, CK, and GPC-1) were considered CTCs. At the same time, a high number of the putative tumor cells significantly positive for one or two of the three antigens was also observed in the patients’ samples, representing potential antigen heterogeneity of the PCa tumor. (**C**) The MRI scan of one of the PCa patients’ prostates (white circle) showing the tumor (red circle) as the potential source of CTCs in the seminal fluid.

As a result, CTCs (Figure 3A) were found in all processed SF samples collected from the patients with diagnosed PCa (Figure 3C). The number of the isolated CTCs varied from 67 to 307 per mL (Table 1), with the median value of 104 and the mean value of 135 cells. However, cells overexpressing CK, PSMA, and GPC-1 were identified in 2 of 15 samples in the healthy donor SF samples, although the number of identified abnormal cells was significantly lower ranging from 1.2 to 3 cells per mL for each of the two samples, with the total number of such cells not exceeding 6 units per sample. Figure 3 shows representative images of the antibody-based fluorescent labelling of CTCs isolated from the patients’ samples. It is worth noting that a significant level of the heterogeneity in the intensity of expression between the different antigens was observed (Figure 3B).

### 3.3. Potential Diagnostic and Prognostic Value of the Method

**Figure 4:**
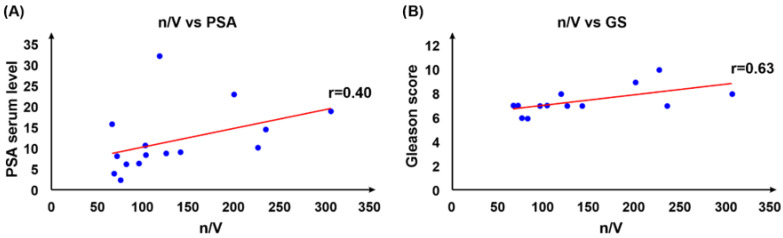

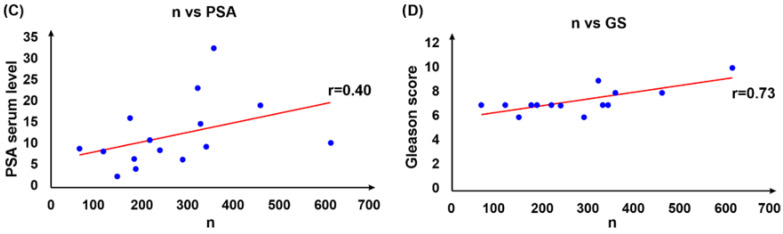
Correlations between n/V vs. PSA (**A**) and n/V vs. GS (**B**); n vs. PSA (**C**) and n vs. GS (**D**). A relatively weak correlation was identified for n/V vs. PSA with r value at r = 0.40, and for n vs. PSA with r value at r = 0.42. At the same time, moderate correlation was identified for n/V vs. GS with r value at r = 0.63, and for n vs. GS with r value at r = 0.73.

The potential diagnostic and prognostic applicability of the developed method was evaluated taking into consideration the absolute number of CTCs (n), the number of CTCs per mL of the SF (n/V), and conventional diagnostic and prognostic parameters such as PSA serum level or GS (Table 1). The correlation coefficients (*r*) for n/V vs. PSA and n/V vs. GS were measured as 0.40 and 0.63, respectively. Furthermore, *r*-values for *n* vs. PSA, *n* vs. GS, and GS vs. PSA were measured as 0.42, 0.73, and 0.51, respectively. Thus, a weak positive correlation was found between the PSA serum level and total numbers of the isolated CTCs, as well as the number of CTCs per mL of the SF, with an insignificant difference between the *r*-values. At the same time, a moderate positive correlation was found between the GS and the total number of the isolated CTCs, as well as the number of CTCs per mL of the SF, with no significant difference between the *r*-values (Figure 4). A moderate correlation was found between the GS and PSA values (Figure S1). Furthermore, a weak positive correlation was identified between the number of the isolated CTCs and the total volume of the SF sample (*r* estimated as 0.33).

## 4. Discussion

During the past decades, in clinical practice, the PSA blood test has been the most trusted tool for the early detection of PCa and its prognostics [2]. Another tool, conventionally used in diagnosing PCa, has been the tissue biopsy. However, both diagnostic tools have significant disadvantages. Thus, PSA blood test has a relatively low specificity, while tissue biopsy has only moderate sensitivity if performed without being accompanied by radiological techniques and may also have serious complications [3]. Recently, in PCa management, liquid biopsy by means of microfluidics-based isolation of CTCs presenting in biological fluids was proposed as the prospective alternative to the existing conventional diagnostic and prognostic techniques [31]. However, according to the published data, liquid biopsy of neither blood [32,33] nor urine [15] demonstrated an outstanding performance in detecting PCa at its early stage. Therefore, investigation of other biological fluids as the potential sources of CTCs, particularly semen, is of great interest.

We tested this hypothesis by employing microfluidic label-free isolation of PCa CTCs from the SF of the patients diagnosed with PCa at its localized form. According to the obtained results, the technique based on inertial microfluidics demonstrated remarkably high efficiency in separating PCa CTCs from sperm cells presenting in the SF. Initially, flow rates ranging from 1.1 to 2.1 mL/min were tested for PCa cell isolation by using healthy SF spiked with PCa cell lines. Due to the particular structure and cross-section of the microchannel, sperm and PCa cells experience two unique forces upon introduction into the microchannel under a continuous fluid flow, which are inertial lift and Dean drag forces. These two forces are strongly dependent on the particle sizes, shape, and morphology. The microchannel of the microfluidic device was designed to focus sperm cells towards one side of the channel and PCa cells towards the opposite side of the channel [34]. In this pilot experiment with healthy SF samples spiked with DU-145and LNCaP cells, it was possible to isolate over 88% of PC3 cells and eliminate more than 99% of the sperm cells. As a result, an optimal flow rate of 1.7 mL/min was indicated and further used for spiking experiments with DU-145, PC3, and LNCaP cells. This approach offers the opportunity to isolate CTCs for early PCa diagnosis and prognostics, and also to purge SF from the tumor cells with the intent of its cryopreservation in men with cancer of reproductive organs. Conventional methods of cancer therapy, including chemotherapy and radiotherapy, often prove detrimental to the fertility potential and spermatogenesis in PCa patients [35]. Furthermore, patients with cancer of the reproductive organs typically present lower-quality sperm with decreased concentration, motility, and DNA integrity [36]. Therefore, the isolation of sperm cells from cancer cells achieved in this study has great potential for bio-banking before treatments are applied to patients. The isolation of sperm cells from cancer cells serves to preserve sperm quality as cancer cells and other epithelial cells can release reactive oxygen species which can induce sperm DNA fragmentation, leading to potentially lower rates of sperm recovery post-cryopreservation and lower rates of fertilized embryo implantation [37].

Following the successful isolation of PCa cell lines from the spiked SF samples with the microfluidic chip using the optimized fluid-flow rate at 1.7 mL/min, the same technique was applied to the SF samples from PCa patients and healthy donors as a control. To ensure high specificity of the technique, an immunocytochemistry panel detecting several PCa-specific antigens, such as anti-CK, anti-PSMA, and anti-GPC-1, was applied for identifying isolated CTCs. All 15 out of 15 patients with localized PCa (100%) had CTCs (CK+, PSMA+, and GPC-1+ cells) in their SF, varying from approximately 66.9 to 306.7 cells per mL and from 63 to 613 units per sample, representing a 20–30-fold improvement in CTC isolation when compared with previously reported studies where CTCs were isolated from blood or urine [32,38]. At the same time, among the SF samples collected from the healthy volunteers, 2 of 15 had 1.2 and 3 abnormal cells (CK+, PSMA+, and GPC-1+ cells) per mL, with the maximal number of the isolated abnormal cells at 6 units per sample. Considering that the patient with the lowest CTC number had 63 cells in his SF sample, all 15 patients had a far higher number of CTCs than both healthy volunteers whose SF samples were positive for the abnormal cells. Therefore, the developed technique demonstrated an outstanding specificity and sensitivity in detecting putative PCa CTCs. Furthermore, the described method of CTC isolation from SF is relatively straightforward and quick, with the sample-processing time at less than 5 min, and without the need for complex equipment, requiring only the microfluidic chip, syringe, and syringe pump.

The correlations between the number of isolated CTCs, PSA serum level, or GS represent another significant result of this study. It is worth noting that, while the correlations between the absolute number of CTCs or the number of CTCs per mL and PSA serum level were modest, a moderate positive correlation between the numbers of CTCs and GS were observed. This indicated that the microfluidic CTC isolation from the SF may potentially have prognostic value in PCa risk stratification and treatment, particularly as an alternative to the PSA test, known for its notoriously poor sensitivity. At the same time, it should be considered that the developed technique has significant limitation based on the impossibility of semen dotation in some elderly men, especially men with erectile dysfunction. Additionally, the proposed technique may potentially be applied for evaluating the expression of different antigens by the isolated CTCs, which may be valuable for decision making during such conventional approaches for treating localized PCa as watchful waiting or active surveillance.

## 5. Conclusions

Overall, the conducted technique demonstrated great potential for being applied as a diagnostic and prognostic tool in PCa management. Notably, the proposed technique allowed the isolation of a significantly higher number of CTCs from the SF in comparison with the techniques, reported in the literature, previously developed for isolating CTCs from blood and urine. To better reveal the diagnostic and prognostic potential of the developed technique, a study involving large cohorts of PCa patients and healthy volunteers is required. Additionally, a longitudinal investigation based on the analysis of multiple semen samples, provided by each PCa patient at different time points, is highly relevant. Furthermore, comparison of diagnostic and prognostic values between diverse antibody panels, used for immunocytochemical identification of the isolated CTCs, is of great interest. It is anticipated that the developed technique may be applicable for isolating CTCs originating from not only PCa but also adenocarcinoma and testicular cancer, as well as for purifying semen from epithelial cells and large debris with the purpose of its further cryopreservation.

## Figures and Tables

**Table 1:** Number of isolated CTCs from SF samples and parameters of PCa in patients.

Patient Number	Age	Tumor Localization (Zone/Lobe)	*n* *	V **	*n*/V	TNM Stage	Gleason Score	PSA Level
1	42	Peripheral /right	217	2.1	103.3	T1cN0M0	7	10.8
2	69	Peripheral/right	321	1.6	200.6	T2cN0M0	9	23
3	56	Peripheral/left	238	2.3	103.5	T2cN0M0	7	8.5
4	61	Peripheral/left and right	289	3.5	82.6	T2cN0M0	6	6.3
5	51	Central	460	1.5	306.7	T1bN0M0	8	19
6	56	Peripheral/right	183	1.9	96.3	T1cN0M0	7	6.5
7	51	Peripheral/right	174	2.6	66.9	T2bN0M0	7	16
8	59	Transitory/left	357	3	119.0	T2bN0M0	8	32.4
9	62	Peripheral/left	187	2.7	69.3	T1cN0M0	7	4.1
10	50	Peripheral/right	63	0.5	126	T1cN0M0	7	8.9
11	67	Peripheral/left	145	1.9	76.3	T1cN0M0	6	2.3
12	70	Transitory/left	329	1.4	235	T1cN0M0	7	14.7
13	63	Peripheral/left	115	1.6	71.9	T1cN0M0	7	8.2
14	70	Peripheral/left	613	2.7	227	T2cN0M0	10	10.2
15	72	Peripheral/right	340	2.4	141.7	T2cN0M0	7	9.2

*—total number of PSMA+, CK+, GPC-1+ putative tumor cells in the patients’ SF samples. **—total volume of a patients’ SF samples.

## Data Availability

The datasets generated during and/or analyzed during the current study are available from the corresponding author A.S.R. on request.

## Supplementary Materials

The following supporting information can be downloaded at: https://www.mdpi.com/article/10.3390/cancers14143364/s1, Figure S1: Correlations between GS and PSA presenting a moderate correlation between the two and an r value of 0.51; Figure S2: Recovery rates of spiked PC3, DU-145, LNCaP, and sperm cells at different flow rates in processed semen from the waste outlet; Figure S3: Recovery rates of spiked PC3, DU-145, and LNCaP at different flow rates in DPBS from the target outlet; Video S1: isolation of spiked PCa tumor cells from a semen sample.
