# Supplementary material for: Isolation of Circulating Tumor Cells from Seminal Fluid of Patients with Prostate Cancer Using Inertial Microfluidics
Source: Cancers (Basel). 2022 Jul 11;14(14):3364. doi: 10.3390/cancers14143364 (PMC9318520; doi:10.3390/cancers14143364)
Supplement: Supplementary file 1 [file cancers-14-03364-s001.zip › cancers-1739869 supplementary/Figures S1-S3.pdf]

Electronic Supplementary Information

Isolation of Circulating Tumor Cells from Seminal Fluid of Patients with Prostate Cancer Using Inertial Microfluidics

Alexey S. Rzhevskiy, Alina Y. Kapitannikova, Steven A. Vasilescu, Tamilla A. Karashaeva, Sajad Razavi Bazaz, Mark S. Taratkin, Dmitry V. Enikeev, Vladimir Yu. Lekarev, Evgeniy V. Shpot, Denis V. Butnaru, Sergey M. Deyev, Jean Paul Thiery, Andrei V. Zvyagin and Majid Ebrahimi Warkiani

This supplementary file includes:

- Gleason score (GS) and prostate specific antigen (PSA) correlation graph

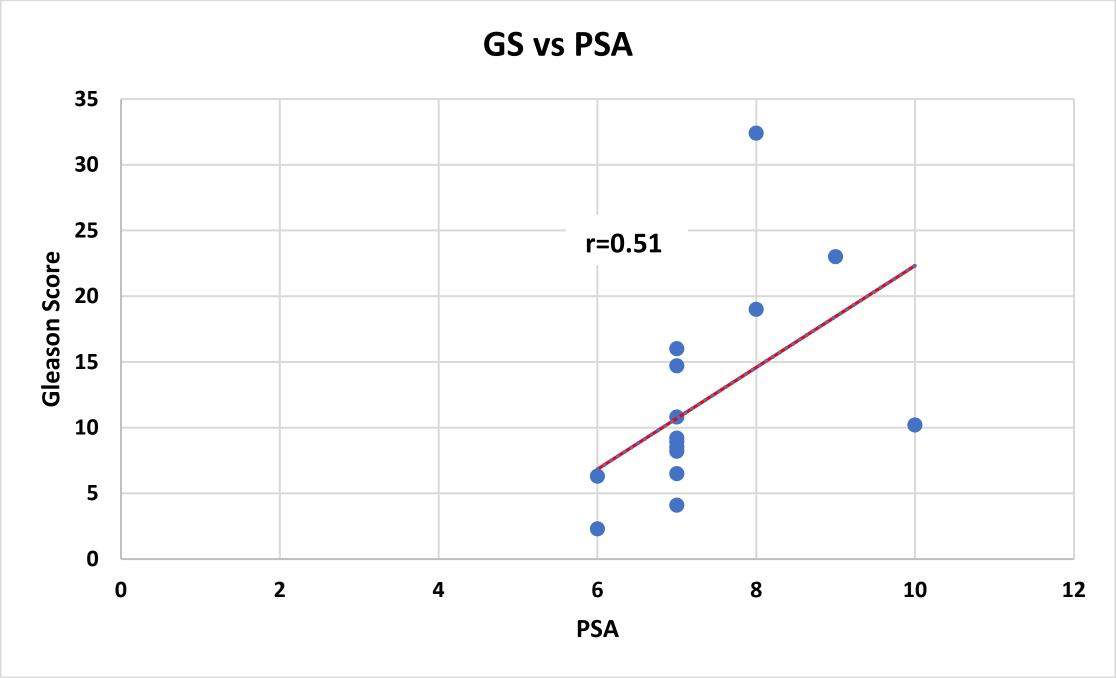

Figure S1. Correlations between GS and PSA presenting a moderate correlation between the two and an r value of 0.51

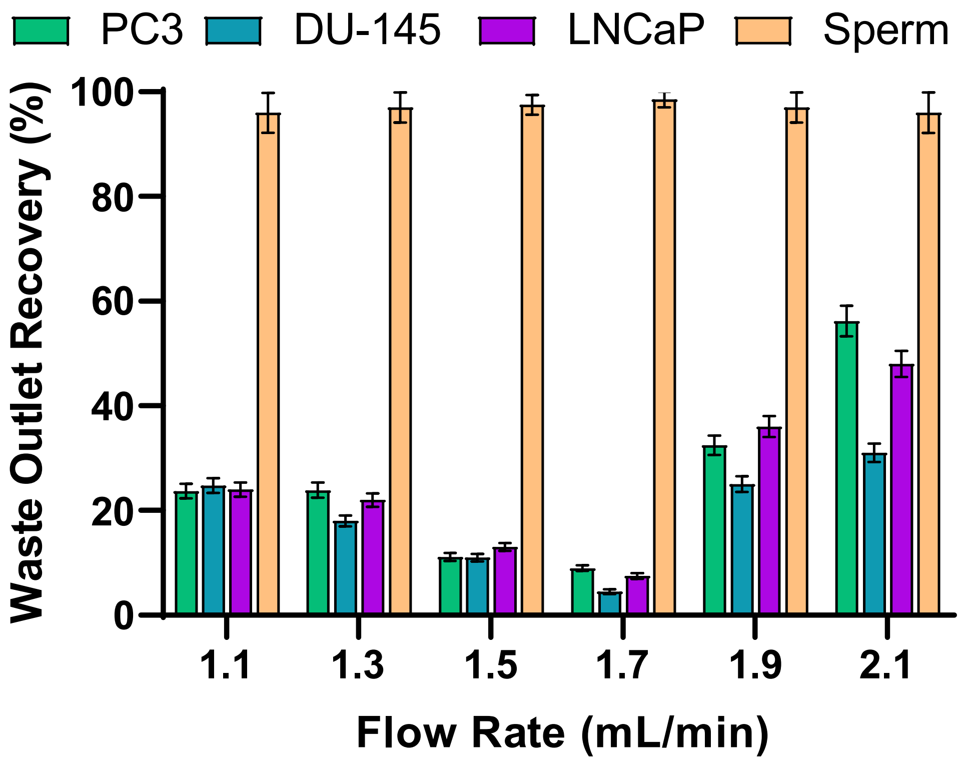

Figure S2. Recovery rates of spiked PC3, DU-145, LNCaP , and sperm cells at different flow rates in processed semen from the waste outlet.

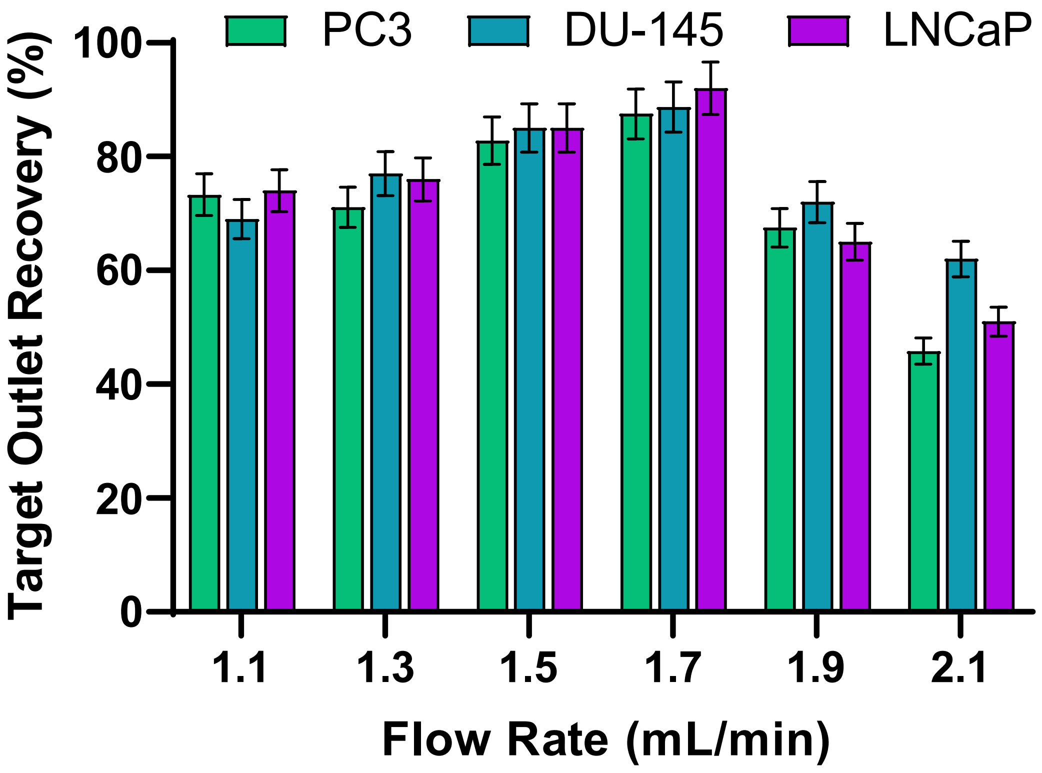

Figure S3. Recovery rates of spiked PC3, DU-145, and LNCaP at different flow rates in DPBS from the target outlet.
